# A new co-crystal dinuclear/trinuclear Zn^II^–Zn^II^/Zn^II^–Sm^III^–Zn^II^ complex with a salen-type Schiff base ligand

**DOI:** 10.1107/S2056989018016109

**Published:** 2018-11-22

**Authors:** Mamour Sarr, Mayoro Diop, Elhadj Ibrahima Thiam, Mohamed Gaye, Aliou Hamady Barry, James B. Orton, Simon J Coles

**Affiliations:** a Département de Chimie, Faculté des Sciences et Techniques, Université Cheikh Anta Diop, Dakar, Senegal; b Département de Chimie, Faculté des Sciences, Université de Nouakchott, Nouakchott, Mauritania; c UK National Crystallography Service, School of Chemistry, Faculty of Engineering and Physical Sciences, University of Southampton, SO17 1BJ, UK

**Keywords:** crystal structure, zinc, samarium, Schiff base

## Abstract

In the penta­nuclear title complex, [SmZn_2_(C_22_H_18_N_2_O_4_)_2_(NCS)_2_(C_3_H_7_NO)_2_][Zn_2_(C_22_H_18_N_2_O_4_)(NCS)_3_]·C_3_H_7_NO·0.32H_2_O, namely bis­{μ_2_-6,6′-dimeth­oxy-2,2′-[phenyl­ene-1,2-diylbis(nitrilo­methanylyl­idene)]diphenolato}-1κ^4^ *O*,*N*,*N*′,*O*′:2κ^3^ *O*,*O*′,*O* ^6^;2κ^3^ *O*,*O*′,*O* ^6^:3κ^4^ *O*,*N*,*N*′,*O*′-bis­(di­methyl­formamide-2κ*O*)di­thiocyanato-1κ*N*,3κ*N*-2-samarium(III)-1,3-dizinc(II) {μ_2_-6,6′-dimeth­oxy-2,2′-[phenyl­ene-1,2-diylbis(nitrilo­methanylyl­idene)]diphenolato-1κ^4^ *O*,*N*,*N*′,*O*′:2κ^2^ *O*,*O*′}trithio­cyan­ato-1κ*N*;2κ^2^ *N*,*N*-dizinc(II) di­methyl­formamide monosolvate 1.32-hydrate, a dinuclear unit and a trinuclear unit co-exist. One of the Zn^II^ centers in the dinuclear unit as well as the two Zn^II^ centers in the trinuclear unit are located in the inner N_2_O_2_ cavity of the ligand and are coordinated to the nitro­gen atom of one thio­cyanate moiety, giving rise to a square-pyramidal geometry. The second Zn^II^ center in the dinuclear unit is coordinated to the two phenolate oxygen atoms of the ligand and to two thio­cyanate groups *via* the nitro­gen atom in a tetra­hedral geometry. The Sm^III^ ion is eight-coordinated by four phenolate O atoms from the two ligand mol­ecules, two meth­oxy O atoms from the two ligand mol­ecules and two O atoms from the DFM solvent mol­ecule. In the dinuclear unit, the two meth­oxy oxygen atoms remain uncoordinated while in the trinuclear unit, for each ligand one meth­oxy oxygen is coordinated and the other one remains uncoordinated. In the crystal, the trinuclear cationic units and dinuclear anionic units are assembled into infinite layers. These layers are held together *via* electrostatic inter­actions, forming a three-dimensional structure. In the dinuclear unit, the C and S atoms of one of the thio­cyanate groups are disordered over two sets of sites in a 0.680 (4)(4):0.320 (4) ratio.

## Abstract 2

In the penta­nuclear title complex, [SmZn_2_(C_22_H_18_N_2_O_4_)_2_(NCS)_2_(C_3_H_7_NO)_2_][Zn_2_(C_22_H_18_N_2_O_4_)(NCS)_3_]·C_3_H_7_NO·0.32H_2_O, a dinuclear unit and a trinuclear unit co-exist. In the crystal, the trinuclear cationic units and dinuclear anionic units are assembled into infinite layers.

## Chemical context   

Over recent years, polyheteronuclear complexes of 3*d* and 4*f* metals have been studied with increasing inter­est by chemists (Cristóvão *et al.*, 2017 ▸; Cristóvão & Miroslaw, 2013 ▸; Ding *et al.*, 2015 ▸; Tian *et al.*, 2012 ▸; Wu & Hou, 2010 ▸). The various structures obtained (Rossi *et al.*, 2018 ▸; Zhou *et al.*, 2015 ▸; Ghosh & Ghosh, 2016 ▸), the physicochemical properties (Cristóvão *et al.*, 2017 ▸) and the potential applications in fields such as luminescence (Zhao *et al.*, 2014 ▸; Zhu *et al.*, 2018 ▸), magneto chemistry (Chesman *et al.*, 2012 ▸; Klokishner & Reu, 2012 ▸), electrochemistry (Yin *et al.*, 2017 ▸) and catalysis (Lan *et al.*, 2018 ▸) have made this chemistry very attractive. These compounds are obtained from Schiff bases, which are organic compounds having several donor sites, which are used to assemble stable structures with transition metal or lanthanide ions. Both the nature of the ligand and the nature of the metal strongly influence the properties of the compound obtained. The Schiff bases obtained by condensation between a di­amine and a well-selected keto-precursor may have two cavities of different dimensions, which can accommodate metal ions of different sizes (Andruh, 2011 ▸; Gao *et al.*, 2012 ▸). The salen-type Schiff base obtained by the condensation of 1,2-di­amino­benzene and *ortho-*vanillin has two cavities of different sizes, viz. N_2_O_2_ and O_2_O_2_. The smaller inner N_2_O_2_ cavity consists of two imino nitro­gen atoms and two phenolato oxygen atoms and can encapsulate 3*d* metal ions. The larger outer O_2_O_2_ cavity consists of two phenolato oxygen atoms and two oxygen atoms from meth­oxy groups and can encapsulate 3*d* ions or lanthanide ions that have a larger ionic radius and prefer oxygen because of their hard-acid characters. By controlling the ratio of the ligand–3*d* metal–4*f* metal, it is possible to synthesize 3*d*–3*d* and 3*d*–4*f*–3*d* complexes. It is in this context that we used the ligand *N*,*N*′-bis­(3-meth­oxy­salicyl­idene)phenyl­ene-1,2-di­amine (H_2_ *L*) to synthesize the Zn–Zn/Zn–Sm–Zn co-crystal whose structure is described herein.
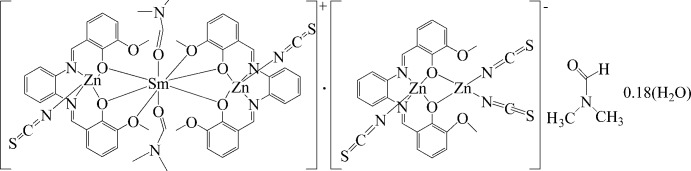


## Structural commentary   

**Figure 1:**
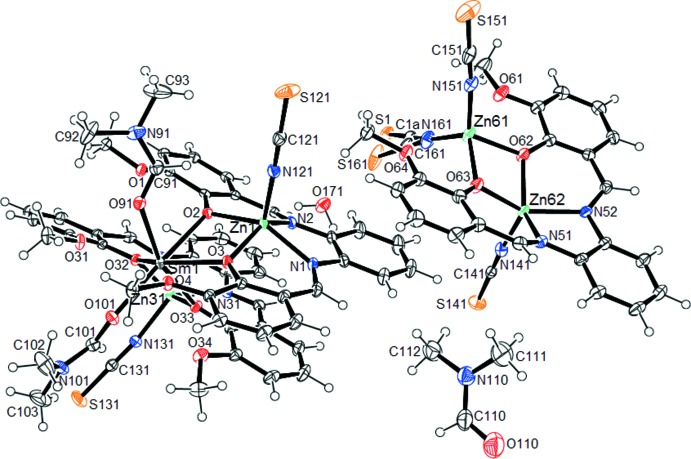
An *ORTEP* view of the asymmetric unit of the title compound, showing the atom-numbering scheme. Displacement ellipsoids are plotted at the 50% probability level.

The title compound crystallizes in the triclinic system in the space group *P*ī. The asymmetric unit (Fig. 1 ▸) consists a co-crystal of one trinuclear cationic unit, [SmZn_2_(*L*)_2_(SCN)_2_(DMF)_2_]^+^, one dinuclear anionic unit, [Zn_2_(*L*)(SCN)_3_]^−^, one uncoordinated DMF solvent mol­ecule and 0.32 of a water mol­ecule.

In the trinuclear unit, both zinc ions are in an N_3_O_2_ environment, which can be characterized by the Addison parameter *τ* [*τ* = (*α* − *β*)/60; *τ* = 0 indicates a regular square-pyramidal geometry and *τ* = 1 indicates a regular trigonal bipyramid; *α* and *β* are the largest angles around the metal ions; Addison *et al.*, 1984 ▸]. The *τ* values of 0.146 for Zn1 and 0.212 for Zn31 are indicative of a severely distorted square-pyramidal geometry around each zinc ion, with the apical positions of each metal ion being occupied by a terminal nitro­gen atom from an anionic thio­cyanate moiety. The apical bond lengths N121—Zn1 and N131—Zn31 are 1.9842 (17) and 1.9786 (16) Å, respectively, and are the shortest distances around these two atoms. The values are slightly lower than those found for the reported Zn_2_Sm complex (Gao *et al.*, 2012 ▸). The equatorial planes around each of these two zinc ions in the trinuclear unit are formed, respectively, by two imino nitro­gen atoms and two phenolate oxygen atoms. The diagonal basal angles, N1—Zn1—O2 = 147.54 (6)° and N2—Zn1—O3 = 138.79 (6)°, N31—Zn31—O32 = 136.30 (6)° and N32—Zn31—O33 = 149.00 (6)° significantly deviate from the ideal values of 180°. The Zn1⋯Zn31, Zn1⋯Sm1 and Sm1⋯Zn31 distances of 5.0288 (5), 3.5372 (5) and 3.5443 (5) Å, respectively, and the Zn1⋯Sm1⋯Zn31, Sm1⋯Zn31⋯Zn1 and Zn31⋯Zn1⋯Sm1 angles of 90.49 (1), 44.70 (1) and 44.81 (1)° respectively, are indicative of an isosceles triangular arrangement of the metal centres in the trinuclear unit.

In the dinuclear unit, all of the meth­oxy oxygen atoms remain uncoordinated, whereas in the trinuclear unit, for each of the two metalloligands, one of the meth­oxy atoms remains uncoordinated (O1 and O34) while the others (O4 and O31) are coordinated to the Sm^III^ atom. The longest bond distances around the Sm^III^ ion are for Sm—O4 [2.6707 (13) Å] and Sm—O31 [2.6934 (13) Å]. The Sm—O_phen­oxy_ distances are in the range 2.3348 (12)–2.4417 (12) Å and are comparable to those found for the Zn_2_Sm complex (Gao *et al.*, 2012 ▸) in which the mean Sm—O_phen­oxy_ distance is 2.332 Å. The Sm—O_DMF_ distances are longer than those found in a samarium complex (Kou *et al.*, 1998 ▸) with Sm—O91 and Sm—O101 values of 2.3831 (13) and 2.3476 (13) Å, respectively (Table 1 ▸). The octa­coordinated polyhedron around the Sm^III^ atom is best described as slightly distorted square anti­prism. The Zn—O_phenoxo_ bond lengths in both the dinuclear and trinuclear units are in the range 1.9985 (13)–2.0395 (12) Å. These values are comparable with the distances for the dinuclear complex [Zn(H_2_O)(valdmpn)Sm(O_2_NO)_3_] [where valdmpn is *N*,*N*′-bis­(3-meth­oxy­salicyl­idene)(2,2-di­methyl­propyl­idene)-1,3-di­amine); Pasatoiu *et al.*, 2012 ▸].

In the trinuclear unit, the Zn(di-μ-phenoxo)_2_Sm bridging fragments show a difference between the Zn—O and Sm—O binding lengths whose mean values are 2.0204 (su?) and 2.3860 (su?) Å, respectively. The four Zn—O_phenoxo_—Sm angles have different values with an averages of 106.54 (su?) and 107.16 (su?)°, respectively, for those involving the Zn1 and Zn31 atoms. The sum of the angles in the Zn1(di-μ-phenoxo)_2_Sm1 and Zn31(di-μ-phenoxo)_2_Sm1 arms are 359.58 and 359.89°, respectively, indicating regular planar geometries. The dihedral angle between Zn1/O2/Sm1/O3 and Zn31/O32/Sm1/O33 plane normals is 76.01 (3)° with the displacement of the respective constituent atoms not exceeding 0.046 and 0.023 Å. In the trinuclear unit, the dihedral angles between the planes O2/Sm1/O3 and O2/Zn1/O3 and the plane normals O32/Sm1/O33 and O32/Zn1/O33 are 6.04 (6) and 3.10 (6)°, respectively. In the dinuclear unit, the dihedral angle between the O62/Zn61/O63 and O62/Zn62/O63 planes is 21.31 (10)°.

In the dinuclear unit, the Zn61 atom is tetra­coordinated while the Zn62 atom is penta­coordinated. The values of the angles around Zn61, which fall in the range 76.86 (6)–119.03 (8)°, are indicative of a distorted tetra­hedral environment. The geometry around the Zn62 atom is best described as a distorted square pyramidal, as indicated by the value of 0.105 for the Addison parameter τ. The apical position is occupied by the nitro­gen atom N141 of the thio­cyanate group with the basal plan occupied by atoms N51, N52, O62 and O63 from the ligand mol­ecule. The angles between the N141 atom in the apical position and each of the four basal plane atoms fall in the range 106.67 (6)–111.37 (7)° and are far from the ideal value of 90°. The deformation of the basal plane around the Zn62 atom is indicated by the values of the *transoid* [138.41 (6) and 144.96 (7)°] and *cisoid* angles [88.34 (6) and 88.94 (6)°], which are different from the ideal values of 180 and 90° for a square-planar geometry (Table 1 ▸). The anionic thio­cyanate ions are N donors and bind to the zinc atoms in a unidentate fashion. The Zn—N—CS bond angles in the dinuclear and trinuclear units are in the range 170.9 (5)–176.12 (18)°, indicating a quasi-linear alignment. The N—C—S angles vary between 177.7 (2) and 179.4 (2)°, showing that these three atoms adopt an almost linear alignment.

## Supra­molecular features   

**Figure 2:**
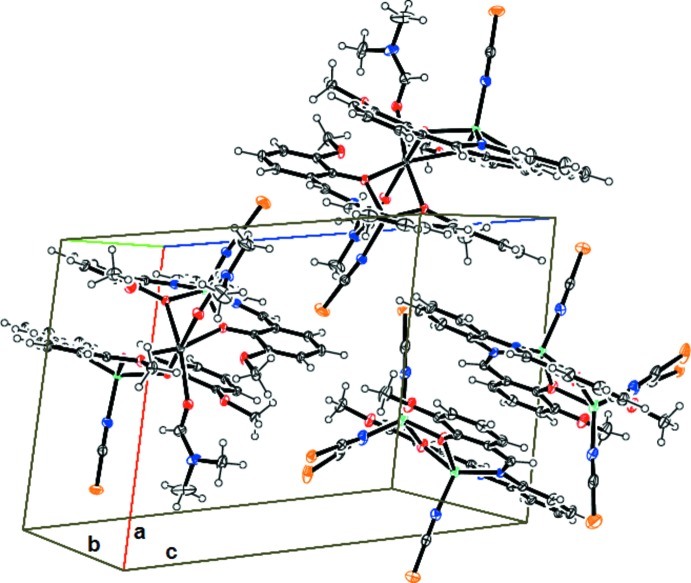
Mol­ecular representation of the title compound, showing the network of dinuclear and trinuclear complex units in layers.

Fig. 2 ▸ shows the packing arrangement in the crystal. The structure is clearly composed of alternating layers composed of cationic units and anionic units stacked along the [101] direction. The complex mol­ecules display no hydrogen-bonding contacts. The trinuclear cationic units and dinuclear anionic units are assembled into infinite layers via electrostatic inter­actions. The alternating ionic layers are held together via electrostatic inter­actions, forming a three-dimensional structure.

## Database survey   

A survey of the Cambridge Structural Database (CSD) (Version 5.39, last update November 2017; Groom *et al.*, 2016 ▸) shows that dinuclear complexes of the ligand bis­(2-hy­droxy-3-meth­oxy­benzyl­idene)-1,2-di­amino­benzene where the smaller N_2_O_2_ cage is occupied by a 3*d* metal and the larger, open O_2_O_2_ cage is occupied by one *s-*, *p-*, *d-* or *f-*block metal are well documented. Trinuclear complexes formed by two 3*d* metals with the above organic ligand in which the 3*d* metal atom is situated in the smaller N_2_O_2_ cage and one *s*-, *d*- or *f*-block metal atom is coordinated to the two larger O_2_O_2_ cages have also been reported: five entries corresponding to *d*–*s* [BIZBAO (Bian *et al.*, 2008 ▸), KAZQEK (Andrez *et al.*, 2017 ▸), KESYOY and KESZAL (Biswas *et al.*, 2013*b* ▸), LARPIG (Feng *et al.*, 2017 ▸)], four entries correspond *d*–*d* [DEDPIM (Yang *et al.*, 2006 ▸), OKECIS (Zhang *et al.*, 2016 ▸), UGAMOF (Wang *et al.*, 2008*b* ▸), WOGQAL (Wang *et al.*, 2008*a* ▸)], seven correspond to *s*–*f* [FEVDUH (Wang *et al.*, 2013 ▸), ITOVIY (Ma *et al.*, 2016 ▸), YIMLUD, YIMMAK, YIMMEO, YIMMIS and YIMMOY (Ma *et al.*, 2013 ▸)], twenty entries correspond to *d*–*f* [AYOKIJ (Yang *et al.*, 2011 ▸), DEJLEK and DEJLAG (Wong *et al.*, 2006 ▸), EBIZUM, EBOBAA and EBOBEE (Chen *et al.*, 2011 ▸), GICBUR and GICCAY (Yang *et al.*, 2013 ▸), KEBGUW (Pushkarev *et al.*, 2017 ▸), MEPXEL, MEPXIP and MEPXOV (Lo *et al.*, 2006 ▸), NOGPIJ (Bi *et al.*, 2008*a* ▸), NOMQIQ, NOMQOW and NOMQUC (Bi *et al.*, 2008*b* ▸), PALZUA (Fu *et al.*, 2017 ▸), POXMIZ (Bi *et al.*, 2009 ▸), VAYBEF (Liu *et al.*, 2017 ▸), YIMMUE (Ma *et al.*, 2013 ▸)], five entries corresponding to *d*–*s*–*d* [DAVZEI (Nandy *et al.*, 2017 ▸), IZEHEB (Das *et al.*, 2011 ▸), KESZEP, KESZIT and KESZOZ (Biswas *et al.*, 2013*a* ▸)], three entries corresponding to *d*–*d*–*d* [DUCJER, DUCJOB and DUCJOB01 (Wang *et al.*, 2009 ▸)]. In all, there are thirteen entries for hetero trinuclear 3*d*–4*f*–3*d* complexes in which the 3*d* metal ion is Zn^2+^ [DEJKUZ and DEJLIO (Wong *et al.*, 2006 ▸), DUCKAO, DUCKOC, DUCKUI, DUCLAP and DUCLET (Wang *et al.*, 2009 ▸), EJAGIG (Liao *et al.*, 2010 ▸), GICCEC and GICCIG (Yang *et al.*, 2013 ▸), QUQKUK, QUQLAR and QUQLEV (Sun *et al.*, 2016 ▸)]. Combinations of mononuclear and hetero dinuclear coordination complexes as co-crystals are observed in three cases [BICBEW and BICBIA (Biswas *et al.*, 2013*b* ▸), KAZPOT (Andrez *et al.*, 2017 ▸)], while the combination of hetero dinuclear and hetero trinuclear coordination complexes as a co-crystal is observed in one case (Sarr *et al.*, 2018 ▸).

## Synthesis and crystallization   

The complex [(Zn*L*)·(H_2_O)] was prepared according to a literature method (Liu *et al.*, 2014 ▸) with slight modification. To a solution of 1,2-di­amino­benzene (0.250 g, 2.31 mmol) in 10 mL of aceto­nitrile was added a solution of *o*-vanillin (0.705 g, 4.62 mmol) in 10 mL of aceto­nitrile. The resulting orange mixture was refluxed for 60 min, affording the organic H_2_ *L* ligand. After cooling, a solution of Zn(CH_3_COO)_2_·2H_2_O (0.507 g, 2.31 mmol) in 10 mL of aceto­nitrile was added. The mixture was heated under reflux for 60 min. On cooling, the orange precipitate was filtered off, washed with 3 × 10 mL of ether and dried in air, yielding a compound formulated as [(ZnL)·(H_2_O)] in 75% yield, m.p. 571–573 K. FT–IR (KBr, ν, cm^−1^): 3307 (OH) (*br*, water), 1609 (C=N) 1594 (C=C), 1586 (C=C), 1488 (C=C), 1439, 1234, 1187, 731. Analysis calculated for C_22_H_20_ZnN_2_O_5_: C, 57.72; H, 4.40; N, 6.12. Found: C, 57.68; H, 4.42; N, 6.07%. Λ (S cm^2^ mol^−1^): 5. The filtrate of a mixture of Sm(NO_3_)_3_·6H_2_O (0.1112 g, 0.25 mmol) and KSCN (0.1458 g, 1.5 mmol) in 20 mL of absolute ethanol was added to a DMF solution (5 mL) of [(ZnL)·(H_2_O)] (0.2288 g, 0.5 mmol). The resulting solution was heated under reflux for two h. After cooling, the solution was filtered and the filtrate was kept at 298 K. After four weeks, crystals suitable for X-ray diffraction were collected and formulated as [{Zn_2_(*L*)(SCN)_3_}]·[Sm{Zn(*L*)(SCN)}_2_(DMF)_2_]·(DMF)·0.18H_2_O. FT–IR (KBr, ν, cm^−1^): 2078 (S=C=N), 1654, 1607 (C=N), 1584 (C=C), 1545 (C=C), 1463 (C=C), 1440, 1238, 1191, 731. Analysis calculated for C_80_H_75.36_Zn_4_SmN_14_O_15.18_S_5_: C, 46.92; H, 3.71; N, 9.57; S, 7.83%. Found: C, C, 46.87; H, 3.68; N, 9.51; S, 7.85%. Λ_M_ (S m^2^ mol^−1^): 28. μ_eff_ = 1.6 µB.

## Refinement   

Crystal data, data collection and structure refinement details are summarized in Table 2 ▸. All H atoms were positioned geometrically (C—H = 0.95–0.98 Å) and refined using a riding model with *U*_iso_(H) = 1.2*U*_eq_(C) or 1.5*U*_eq_(C_meth­yl_, O). One of the thio­cyanate groups was found to be partially disordered such that the C and S atoms 136 of this group were distributed over two positions. In the dinuclear unit, the C and S atoms of one of the thio­cyanate groups are disordered over two sets of sites in a 0.680 (4):0.320 (4) ratio. The water mol­ecule is partially occupied [0.32 (4)].

## Supplementary Material

Crystal structure: contains datablock(s) I. DOI: 10.1107/S2056989018016109/ex2013sup1.cif

Structure factors: contains datablock(s) I. DOI: 10.1107/S2056989018016109/ex2013Isup2.hkl

CCDC reference: 1878960

Additional supporting information:  crystallographic information; 3D view; checkCIF report

## Figures and Tables

**Table 1:** Selected geometric parameters (Å, °)

N1—Zn1	2.0863 (16)	O3—Zn1	1.9985 (13)
N2—Zn1	2.0451 (16)	O4—Sm1	2.6707 (13)
N31—Zn31	2.0534 (16)	O31—Sm1	2.6934 (13)
N32—Zn31	2.0644 (16)	O32—Sm1	2.3599 (12)
N121—Zn1	1.9842 (17)	O32—Zn31	2.0143 (13)
N131—Zn31	1.9786 (16)	O33—Sm1	2.4038 (13)
O2—Sm1	2.4417 (12)	O33—Zn31	2.0175 (13)
O2—Zn1	2.0395 (12)	O91—Sm1	2.3831 (13)
O3—Sm1	2.3348 (12)	O101—Sm1	2.3476 (13)
O2—Sm1—O4	119.60 (4)	O32—Sm1—O31	59.85 (4)
O2—Sm1—O31	111.28 (4)	O32—Sm1—O33	65.80 (4)
O3—Sm1—O2	66.10 (4)	O91—Sm1—O2	83.29 (4)
O3—Sm1—O4	60.69 (4)	O91—Sm1—O4	70.36 (4)
O3—Sm1—O31	159.39 (4)	O91—Sm1—O31	69.71 (4)
O3—Sm1—O32	134.80 (4)	O91—Sm1—O33	162.88 (5)
O3—Sm1—O33	80.48 (4)	N121—Zn1—N1	108.05 (7)
O32—Sm1—O2	78.61 (4)	N121—Zn1—N2	112.95 (7)
O32—Sm1—O4	161.77 (4)	N121—Zn1—O2	104.31 (6)

**Table 2:** Experimental details

Crystal data	
Chemical formula	[Zn_2_(C_52_H_50_N_8_O_10_S_2_Sm)][(Zn_2_(C_25_H_18_N_5_O_4_S_3_)]·C_3_H_7_NO·0.32H_2_O
*M*_r_	2050.43
Crystal system, space group	Triclinic, *P* 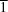
Temperature (K)	100
*a*, *b*, *c* (Å)	14.76937 (9), 15.57623 (10), 19.28129 (13)
α, β, γ (°)	94.7754 (5), 104.1999 (6), 100.9287 (5)
*V* (Å^3^)	4182.75 (5)
*Z*	2
Radiation type	Mo *K*α
μ (mm^−1^)	2.02
Crystal size (mm)	0.33 × 0.21 × 0.17

Data collection	
Diffractometer	Rigaku FRE+ equipped with VHF Varimax confocal mirrors, an AFC12 goniometer and HyPix 6000 detector
Absorption correction	Gaussian (*CrysAlis PRO*; Rigaku OD, 2018 ▸)
*T*_min_, *T*_max_	0.317, 1.000
No. of measured, independent and observed [*I* > 2σ(*I*)] reflections	381624, 19170, 18483
*R*_int_	0.031
(sin θ/λ)_max_ (Å^−1^)	0.649

Refinement	
*R*[*F* ^2^ > 2σ(*F* ^2^)], *wR*(*F* ^2^), *S*	0.024, 0.057, 1.06
No. of reflections	19170
No. of parameters	1115
No. of restraints	14
H-atom treatment	H-atom parameters constrained
Δρ_max_, Δρ_min_ (e Å^−3^)	1.48, −1.13

Computer programs: *CrysAlis PRO* (Rigaku OD, 2018 ▸), *SHELXT* (Sheldrick, 2015*a* ▸), *SHELXL* (Sheldrick, 2015*b* ▸) and *OLEX2* (Dolomanov *et al.*, 2009 ▸).

## supplementary crystallographic information

### Crystal data

**Table:**

[Zn_2_(C_52_H_50_N_8_O_10_S_2_Sm)][(Zn_2_(C_25_H_18_N_5_O_4_S_3_)]·C_3_H_7_NO·0.32H_2_O	*Z* = 2
*M**_r_* = 2050.43	*F*(000) = 2076
Triclinic, *P*1	*D*_x_ = 1.628 Mg m^−^^3^
*a* = 14.76937 (9) Å	Mo *K*α radiation, λ = 0.71075 Å
*b* = 15.57623 (10) Å	Cell parameters from 217952 reflections
*c* = 19.28129 (13) Å	θ = 2.2–31.9°
α = 94.7754 (5)°	µ = 2.02 mm^−^^1^
β = 104.1999 (6)°	*T* = 100 K
γ = 100.9287 (5)°	Block, orange
*V* = 4182.75 (5) Å^3^	0.33 × 0.21 × 0.17 mm

### Data collection

**Table:**

Rigaku FRE+ equipped with VHF Varimax confocal mirrors, an AFC12 goniometer and HyPix 6000 detector diffractometer	19170 independent reflections
Radiation source: Rotating Anode, Rigaku FRE+	18483 reflections with *I* > 2σ(*I*)
Confocal mirrors, VHF Varimax monochromator	*R*_int_ = 0.031
Detector resolution: 10 pixels mm^-1^	θ_max_ = 27.5°, θ_min_ = 1.9°
profile data from ω–scans	*h* = −19→19
Absorption correction: gaussian (CrysAlisPro; Rigaku OD, 2018)	*k* = −20→20
*T*_min_ = 0.317, *T*_max_ = 1.000	*l* = −25→25
381624 measured reflections	

### Refinement

**Table:**

Refinement on *F*^2^	Primary atom site location: dual
Least-squares matrix: full	Hydrogen site location: mixed
*R*[*F*^2^ > 2σ(*F*^2^)] = 0.024	H-atom parameters constrained
*wR*(*F*^2^) = 0.057	*w* = 1/[σ^2^(*F*_o_^2^) + (0.0211*P*)^2^ + 5.627*P*] where *P* = (*F*_o_^2^ + 2*F*_c_^2^)/3
*S* = 1.06	(Δ/σ)_max_ = 0.002
19170 reflections	Δρ_max_ = 1.48 e Å^−^^3^
1115 parameters	Δρ_min_ = −1.13 e Å^−^^3^
14 restraints	

### Special details

**Table:**

Geometry. All esds (except the esd in the dihedral angle between two l.s. planes) are estimated using the full covariance matrix. The cell esds are taken into account individually in the estimation of esds in distances, angles and torsion angles; correlations between esds in cell parameters are only used when they are defined by crystal symmetry. An approximate (isotropic) treatment of cell esds is used for estimating esds involving l.s. planes.
Refinement. One of the thiocyanate ligands is partially disordered. Atoms C161 and S161 are modelled over two positions using thermal parameter restraints. Water molecule O171 partially occupies its site. Its occupancy was refined to 32%, linked to the minor component of the partially disordered thiocyanate.

### Fractional atomic coordinates and isotropic or equivalent isotropic displacement parameters (Å^2^)

**Table:**

	*x*	*y*	*z*	*U*_iso_*/*U*_eq_	Occ. (<1)
C1	0.47788 (14)	0.28188 (13)	1.04840 (11)	0.0234 (4)	
H1A	0.491216	0.223269	1.039509	0.035*	
H1B	0.440589	0.281503	1.084026	0.035*	
H1C	0.538343	0.325565	1.066878	0.035*	
C2	0.35372 (12)	0.40497 (12)	0.91731 (10)	0.0163 (3)	
C3	0.39956 (13)	0.38428 (12)	0.98574 (10)	0.0173 (3)	
C4	0.41594 (13)	0.44118 (13)	1.04845 (10)	0.0202 (4)	
H4	0.447296	0.425959	1.093454	0.024*	
C5	0.38637 (14)	0.52140 (13)	1.04576 (11)	0.0226 (4)	
H5	0.395746	0.559797	1.089062	0.027*	
C6	0.34397 (13)	0.54428 (12)	0.98067 (11)	0.0203 (4)	
H6	0.323774	0.598652	0.979282	0.024*	
C7	0.32954 (12)	0.48859 (12)	0.91529 (10)	0.0175 (3)	
C8	0.29304 (13)	0.52442 (12)	0.85001 (10)	0.0192 (4)	
H8	0.266496	0.574909	0.854613	0.023*	
C9	0.26062 (14)	0.53571 (13)	0.72522 (11)	0.0231 (4)	
C10	0.25789 (18)	0.62528 (15)	0.72943 (13)	0.0338 (5)	
H10	0.277159	0.661216	0.775038	0.041*	
C11	0.2271 (2)	0.66125 (16)	0.66710 (14)	0.0393 (6)	
H11	0.226179	0.722250	0.669859	0.047*	
C12	0.19754 (18)	0.60888 (16)	0.60061 (13)	0.0352 (5)	
H12	0.174177	0.633715	0.558188	0.042*	
C13	0.20176 (16)	0.52053 (15)	0.59538 (12)	0.0291 (4)	
H13	0.181865	0.485229	0.549469	0.035*	
C14	0.23524 (14)	0.48334 (13)	0.65759 (11)	0.0225 (4)	
C15	0.21799 (14)	0.33654 (14)	0.60309 (10)	0.0230 (4)	
H15	0.186779	0.353629	0.558842	0.028*	
C16	0.22727 (14)	0.24583 (13)	0.60267 (10)	0.0213 (4)	
C17	0.20302 (15)	0.19239 (15)	0.53515 (10)	0.0253 (4)	
H17	0.185879	0.217950	0.492077	0.030*	
C18	0.20380 (15)	0.10436 (15)	0.53072 (11)	0.0260 (4)	
H18	0.188572	0.069887	0.484727	0.031*	
C19	0.22688 (14)	0.06467 (13)	0.59352 (10)	0.0218 (4)	
H19	0.227046	0.003518	0.590431	0.026*	
C20	0.24932 (13)	0.11582 (12)	0.65979 (10)	0.0181 (3)	
C21	0.25185 (13)	0.20688 (12)	0.66623 (10)	0.0178 (3)	
C22	0.27917 (16)	−0.00449 (13)	0.72560 (11)	0.0246 (4)	
H22A	0.299045	−0.016821	0.775448	0.037*	
H22B	0.328112	−0.012850	0.700909	0.037*	
H22C	0.218325	−0.044851	0.700303	0.037*	
C31	0.32608 (16)	0.03342 (14)	0.96039 (12)	0.0272 (4)	
H31A	0.334181	0.013293	0.913207	0.041*	
H31B	0.281165	−0.012632	0.974032	0.041*	
H31C	0.388050	0.045749	0.996648	0.041*	
C32	0.23365 (13)	0.22423 (12)	1.00721 (10)	0.0173 (3)	
C33	0.27561 (13)	0.15082 (13)	1.01867 (10)	0.0194 (4)	
C34	0.30241 (14)	0.12524 (14)	1.08608 (11)	0.0237 (4)	
H34	0.327948	0.073914	1.091930	0.028*	
C35	0.29158 (15)	0.17583 (15)	1.14596 (11)	0.0266 (4)	
H35	0.308912	0.158332	1.192586	0.032*	
C36	0.25608 (14)	0.25030 (14)	1.13726 (10)	0.0254 (4)	
H36	0.251603	0.285339	1.178524	0.030*	
C37	0.22583 (13)	0.27649 (13)	1.06822 (10)	0.0202 (4)	
C38	0.19608 (14)	0.35980 (13)	1.06429 (10)	0.0226 (4)	
H38	0.210236	0.399065	1.107828	0.027*	
C39	0.13107 (13)	0.46871 (13)	1.00127 (11)	0.0219 (4)	
C40	0.14305 (15)	0.53108 (14)	1.06130 (13)	0.0288 (4)	
H40	0.164081	0.516947	1.108763	0.035*	
C41	0.12407 (16)	0.61338 (15)	1.05102 (14)	0.0337 (5)	
H41	0.131615	0.655590	1.091666	0.040*	
C42	0.09435 (16)	0.63475 (15)	0.98244 (14)	0.0345 (5)	
H42	0.083192	0.692073	0.976254	0.041*	
C43	0.08054 (15)	0.57354 (14)	0.92236 (13)	0.0295 (4)	
H43	0.059203	0.588534	0.875224	0.035*	
C44	0.09821 (13)	0.48952 (13)	0.93150 (11)	0.0225 (4)	
C45	0.06749 (14)	0.43569 (13)	0.80713 (11)	0.0230 (4)	
H45	0.065584	0.494387	0.797918	0.028*	
C46	0.05095 (14)	0.36976 (13)	0.74565 (11)	0.0220 (4)	
C47	0.00884 (16)	0.39196 (15)	0.67714 (12)	0.0292 (4)	
H47	−0.001569	0.449921	0.673269	0.035*	
C48	−0.01701 (16)	0.33123 (17)	0.61657 (12)	0.0332 (5)	
H48	−0.045509	0.347113	0.571068	0.040*	
C49	−0.00170 (15)	0.24575 (16)	0.62125 (11)	0.0290 (4)	
H49	−0.021575	0.203148	0.579247	0.035*	
C50	0.04234 (14)	0.22333 (14)	0.68703 (10)	0.0222 (4)	
C51	0.07168 (13)	0.28513 (13)	0.75038 (10)	0.0186 (4)	
C52	0.01779 (16)	0.07149 (16)	0.64128 (13)	0.0334 (5)	
H52A	0.041190	0.083384	0.598912	0.050*	
H52B	−0.051725	0.065734	0.628769	0.050*	
H52C	0.033356	0.016528	0.656932	0.050*	
C61	0.5946 (2)	1.04315 (18)	0.77533 (12)	0.0451 (7)	
H61A	0.651492	1.087975	0.776329	0.068*	
H61B	0.613469	0.998703	0.805712	0.068*	
H61C	0.549523	1.071190	0.793689	0.068*	
C62	0.45184 (14)	1.00189 (13)	0.58964 (10)	0.0207 (4)	
C63	0.51311 (14)	1.05321 (14)	0.65420 (11)	0.0239 (4)	
C64	0.53124 (15)	1.14368 (14)	0.66317 (12)	0.0271 (4)	
H64	0.569312	1.176545	0.707936	0.033*	
C65	0.49331 (16)	1.18724 (14)	0.60597 (12)	0.0285 (4)	
H65	0.504546	1.249856	0.612180	0.034*	
C66	0.43987 (15)	1.13970 (14)	0.54088 (12)	0.0261 (4)	
H66	0.418122	1.169986	0.501405	0.031*	
C67	0.41665 (14)	1.04641 (13)	0.53161 (11)	0.0215 (4)	
C68	0.35884 (14)	1.00181 (13)	0.46112 (11)	0.0222 (4)	
H68	0.351414	1.035837	0.422254	0.027*	
C69	0.26370 (14)	0.87812 (14)	0.37728 (10)	0.0226 (4)	
C70	0.22243 (16)	0.92248 (16)	0.32200 (11)	0.0293 (4)	
H70	0.229898	0.984743	0.329941	0.035*	
C71	0.17050 (17)	0.87523 (17)	0.25547 (12)	0.0344 (5)	
H71	0.142465	0.905339	0.217773	0.041*	
C72	0.15917 (16)	0.78435 (16)	0.24355 (12)	0.0319 (5)	
H72	0.123988	0.752733	0.197577	0.038*	
C73	0.19858 (14)	0.73949 (15)	0.29790 (11)	0.0265 (4)	
H73	0.190194	0.677168	0.289380	0.032*	
C74	0.25078 (14)	0.78568 (14)	0.36542 (10)	0.0224 (4)	
C75	0.30916 (14)	0.66808 (14)	0.41934 (11)	0.0244 (4)	
H75	0.294017	0.637218	0.371930	0.029*	
C76	0.35154 (15)	0.62422 (14)	0.47861 (11)	0.0244 (4)	
C77	0.35354 (16)	0.53432 (15)	0.46215 (12)	0.0304 (5)	
H77	0.327367	0.505985	0.413826	0.036*	
C78	0.39237 (17)	0.48743 (15)	0.51437 (13)	0.0343 (5)	
H78	0.390680	0.426635	0.502367	0.041*	
C79	0.43454 (16)	0.52873 (14)	0.58538 (13)	0.0302 (5)	
H79	0.462072	0.496328	0.621501	0.036*	
C80	0.43585 (15)	0.61642 (14)	0.60247 (11)	0.0257 (4)	
C81	0.39344 (14)	0.66613 (13)	0.55010 (11)	0.0239 (4)	
C82	0.52080 (18)	0.62428 (16)	0.72639 (13)	0.0365 (5)	
H82A	0.573459	0.602533	0.713608	0.055*	
H82B	0.474809	0.574719	0.734843	0.055*	
H82C	0.546158	0.667221	0.770334	0.055*	
C91	0.48768 (15)	0.21332 (13)	0.86469 (11)	0.0244 (4)	
H91	0.487373	0.264835	0.841573	0.029*	
C92	0.5778 (2)	0.1232 (2)	0.93742 (13)	0.0433 (7)	
H92A	0.592493	0.142763	0.989351	0.065*	
H92B	0.628646	0.095895	0.927387	0.065*	
H92C	0.516687	0.079954	0.921885	0.065*	
C93	0.66121 (18)	0.25655 (18)	0.9000 (2)	0.0573 (9)	
H93A	0.648782	0.303536	0.870560	0.086*	
H93B	0.701183	0.222644	0.880642	0.086*	
H93C	0.694468	0.282697	0.949917	0.086*	
C101	0.07480 (14)	0.02528 (14)	0.84680 (11)	0.0247 (4)	
H101	0.043799	0.067091	0.864475	0.030*	
C102	0.0824 (2)	−0.12585 (17)	0.81992 (19)	0.0544 (8)	
H10A	0.040705	−0.157291	0.773556	0.082*	
H10B	0.090995	−0.167595	0.855051	0.082*	
H10C	0.144704	−0.098456	0.813532	0.082*	
C103	−0.0466 (2)	−0.0882 (2)	0.86993 (17)	0.0514 (7)	
H10D	−0.067862	−0.036988	0.888074	0.077*	
H10E	−0.032022	−0.124630	0.908563	0.077*	
H10F	−0.097489	−0.123217	0.829274	0.077*	
C110	−0.0162 (2)	0.34916 (18)	0.28550 (16)	0.0479 (7)	
H110	−0.054878	0.326883	0.315544	0.057*	
C111	0.1395 (3)	0.4214 (3)	0.2762 (2)	0.0765 (12)	
H11A	0.199011	0.400565	0.291166	0.115*	
H11B	0.153809	0.486088	0.284269	0.115*	
H11C	0.109420	0.400721	0.224752	0.115*	
C112	0.1110 (2)	0.3983 (2)	0.39459 (16)	0.0572 (8)	
H11D	0.162972	0.366962	0.407982	0.086*	
H11E	0.058861	0.373820	0.415257	0.086*	
H11F	0.134829	0.461119	0.413244	0.086*	
C121	0.54729 (15)	0.41108 (14)	0.75563 (10)	0.0245 (4)	
C131	−0.07003 (13)	0.16694 (13)	0.93407 (10)	0.0202 (4)	
C141	0.13318 (15)	0.82477 (13)	0.56942 (10)	0.0228 (4)	
C151	0.69896 (19)	0.82208 (17)	0.66168 (11)	0.0350 (5)	
C161	0.4141 (6)	0.8390 (5)	0.7919 (4)	0.0362 (17)	0.680 (4)
C961	0.4396 (10)	0.8561 (10)	0.7940 (8)	0.020 (2)	0.320 (4)
N1	0.24878 (12)	0.39575 (11)	0.65877 (9)	0.0203 (3)	
N2	0.29349 (11)	0.49364 (10)	0.78619 (9)	0.0195 (3)	
N31	0.08461 (11)	0.42159 (10)	0.87339 (9)	0.0194 (3)	
N32	0.15182 (11)	0.38385 (10)	1.00570 (9)	0.0200 (3)	
N51	0.29036 (12)	0.74530 (11)	0.42560 (9)	0.0214 (3)	
N52	0.31698 (11)	0.91976 (11)	0.44740 (9)	0.0208 (3)	
N91	0.57117 (13)	0.19849 (12)	0.89834 (10)	0.0289 (4)	
N101	0.03909 (13)	−0.05827 (12)	0.84590 (10)	0.0296 (4)	
N110	0.07606 (17)	0.38786 (15)	0.31729 (12)	0.0433 (5)	
N121	0.46702 (12)	0.39695 (11)	0.75462 (10)	0.0245 (3)	
N131	−0.01118 (11)	0.21749 (10)	0.92008 (9)	0.0195 (3)	
N141	0.20378 (13)	0.82501 (12)	0.55333 (9)	0.0258 (4)	
N151	0.62234 (14)	0.83099 (13)	0.65747 (10)	0.0305 (4)	
N161	0.45048 (16)	0.83626 (13)	0.74264 (10)	0.0349 (4)	
O1	0.42459 (9)	0.30425 (9)	0.98237 (7)	0.0192 (3)	
O2	0.33573 (9)	0.34532 (8)	0.85947 (7)	0.0166 (2)	
O3	0.27275 (9)	0.24984 (8)	0.73285 (7)	0.0175 (2)	
O4	0.26748 (10)	0.08498 (8)	0.72583 (7)	0.0192 (3)	
O31	0.28939 (10)	0.11197 (9)	0.95643 (7)	0.0216 (3)	
O32	0.20855 (9)	0.24163 (8)	0.94012 (7)	0.0166 (2)	
O33	0.11769 (9)	0.26091 (9)	0.81137 (7)	0.0183 (3)	
O34	0.06243 (10)	0.14280 (10)	0.69857 (8)	0.0251 (3)	
O61	0.55042 (12)	1.00204 (10)	0.70337 (8)	0.0321 (3)	
O62	0.43286 (10)	0.91519 (9)	0.58715 (7)	0.0248 (3)	
O63	0.39607 (11)	0.74948 (9)	0.57098 (8)	0.0268 (3)	
O64	0.47437 (12)	0.66543 (10)	0.66895 (8)	0.0304 (3)	
O91	0.40942 (10)	0.16447 (9)	0.86118 (7)	0.0212 (3)	
O101	0.14673 (10)	0.05369 (9)	0.82604 (8)	0.0239 (3)	
O110	−0.05398 (19)	0.34068 (16)	0.21991 (13)	0.0669 (6)	
O971	0.2471 (4)	0.7241 (3)	0.8914 (3)	0.0312 (13)	0.320 (4)
H97A	0.191678	0.713560	0.859708	0.047*	0.320 (4)
H97B	0.286528	0.765421	0.879411	0.047*	0.320 (4)
S121	0.66030 (4)	0.43263 (6)	0.75822 (3)	0.04730 (18)	
S131	−0.15358 (5)	0.09592 (4)	0.95208 (4)	0.04343 (15)	
S141	0.03489 (4)	0.82561 (4)	0.59226 (4)	0.03809 (13)	
S151	0.80856 (6)	0.81383 (8)	0.66793 (4)	0.0716 (3)	
S161	0.36571 (19)	0.84272 (15)	0.85955 (6)	0.0601 (7)	0.680 (4)
S961	0.41791 (18)	0.88705 (18)	0.86980 (10)	0.0261 (6)	0.320 (4)
Sm1	0.25154 (2)	0.19066 (2)	0.83656 (2)	0.01485 (3)	
Zn1	0.33194 (2)	0.37866 (2)	0.75889 (2)	0.01671 (4)	
Zn31	0.09979 (2)	0.30267 (2)	0.90801 (2)	0.01559 (4)	
Zn61	0.48983 (2)	0.83366 (2)	0.65355 (2)	0.02684 (5)	
Zn62	0.31979 (2)	0.83012 (2)	0.51943 (2)	0.02048 (5)	

### Atomic displacement parameters (Å^2^)

**Table:**

	*U*^11^	*U*^22^	*U*^33^	*U*^12^	*U*^13^	*U*^23^
C1	0.0196 (9)	0.0251 (10)	0.0234 (9)	0.0034 (7)	0.0012 (7)	0.0090 (8)
C2	0.0123 (8)	0.0167 (8)	0.0186 (8)	−0.0008 (6)	0.0045 (6)	0.0024 (7)
C3	0.0137 (8)	0.0171 (8)	0.0203 (9)	0.0007 (6)	0.0043 (7)	0.0039 (7)
C4	0.0166 (9)	0.0232 (9)	0.0179 (9)	0.0004 (7)	0.0023 (7)	0.0026 (7)
C5	0.0207 (9)	0.0226 (9)	0.0217 (9)	−0.0001 (7)	0.0058 (7)	−0.0028 (7)
C6	0.0176 (9)	0.0164 (8)	0.0263 (10)	0.0015 (7)	0.0070 (7)	0.0008 (7)
C7	0.0120 (8)	0.0178 (8)	0.0214 (9)	0.0006 (6)	0.0041 (7)	0.0029 (7)
C8	0.0147 (8)	0.0160 (8)	0.0264 (9)	0.0016 (7)	0.0052 (7)	0.0050 (7)
C9	0.0240 (10)	0.0222 (9)	0.0266 (10)	0.0066 (8)	0.0093 (8)	0.0120 (8)
C10	0.0479 (14)	0.0233 (10)	0.0322 (12)	0.0106 (10)	0.0100 (10)	0.0101 (9)
C11	0.0564 (16)	0.0257 (11)	0.0414 (13)	0.0165 (11)	0.0133 (12)	0.0172 (10)
C12	0.0456 (14)	0.0345 (12)	0.0338 (12)	0.0181 (10)	0.0133 (10)	0.0212 (10)
C13	0.0358 (12)	0.0325 (11)	0.0253 (10)	0.0139 (9)	0.0115 (9)	0.0142 (9)
C14	0.0230 (9)	0.0246 (10)	0.0254 (10)	0.0082 (8)	0.0110 (8)	0.0130 (8)
C15	0.0263 (10)	0.0294 (10)	0.0183 (9)	0.0111 (8)	0.0084 (8)	0.0118 (8)
C16	0.0232 (9)	0.0265 (10)	0.0167 (9)	0.0087 (8)	0.0064 (7)	0.0060 (7)
C17	0.0275 (10)	0.0363 (11)	0.0157 (9)	0.0130 (9)	0.0065 (8)	0.0066 (8)
C18	0.0278 (10)	0.0358 (11)	0.0156 (9)	0.0116 (9)	0.0058 (8)	−0.0001 (8)
C19	0.0221 (9)	0.0242 (9)	0.0203 (9)	0.0075 (7)	0.0068 (7)	0.0006 (7)
C20	0.0174 (8)	0.0227 (9)	0.0157 (8)	0.0059 (7)	0.0055 (7)	0.0044 (7)
C21	0.0158 (8)	0.0231 (9)	0.0152 (8)	0.0046 (7)	0.0054 (7)	0.0034 (7)
C22	0.0337 (11)	0.0204 (9)	0.0227 (9)	0.0112 (8)	0.0079 (8)	0.0051 (7)
C31	0.0365 (12)	0.0235 (10)	0.0281 (10)	0.0128 (9)	0.0128 (9)	0.0127 (8)
C32	0.0142 (8)	0.0209 (9)	0.0151 (8)	−0.0010 (7)	0.0044 (6)	0.0032 (7)
C33	0.0182 (9)	0.0224 (9)	0.0162 (8)	−0.0006 (7)	0.0049 (7)	0.0051 (7)
C34	0.0227 (10)	0.0272 (10)	0.0201 (9)	0.0010 (8)	0.0047 (7)	0.0105 (8)
C35	0.0248 (10)	0.0368 (11)	0.0152 (9)	−0.0005 (8)	0.0038 (7)	0.0089 (8)
C36	0.0236 (10)	0.0349 (11)	0.0147 (9)	−0.0006 (8)	0.0056 (7)	0.0013 (8)
C37	0.0163 (9)	0.0255 (9)	0.0162 (8)	−0.0006 (7)	0.0040 (7)	0.0011 (7)
C38	0.0195 (9)	0.0268 (10)	0.0194 (9)	0.0008 (7)	0.0070 (7)	−0.0049 (7)
C39	0.0132 (8)	0.0196 (9)	0.0311 (10)	0.0003 (7)	0.0073 (7)	−0.0037 (8)
C40	0.0210 (10)	0.0288 (11)	0.0335 (11)	0.0024 (8)	0.0075 (8)	−0.0077 (9)
C41	0.0241 (11)	0.0268 (11)	0.0467 (14)	0.0030 (8)	0.0110 (10)	−0.0134 (10)
C42	0.0271 (11)	0.0215 (10)	0.0552 (15)	0.0063 (8)	0.0143 (10)	−0.0046 (10)
C43	0.0240 (10)	0.0240 (10)	0.0418 (12)	0.0064 (8)	0.0111 (9)	0.0017 (9)
C44	0.0136 (8)	0.0193 (9)	0.0336 (11)	0.0008 (7)	0.0085 (8)	−0.0020 (8)
C45	0.0185 (9)	0.0201 (9)	0.0315 (10)	0.0044 (7)	0.0069 (8)	0.0081 (8)
C46	0.0180 (9)	0.0271 (10)	0.0223 (9)	0.0051 (7)	0.0061 (7)	0.0092 (8)
C47	0.0287 (11)	0.0354 (11)	0.0287 (11)	0.0141 (9)	0.0082 (9)	0.0160 (9)
C48	0.0309 (11)	0.0510 (14)	0.0221 (10)	0.0184 (10)	0.0046 (9)	0.0156 (10)
C49	0.0239 (10)	0.0454 (13)	0.0178 (9)	0.0115 (9)	0.0032 (8)	0.0031 (9)
C50	0.0182 (9)	0.0305 (10)	0.0201 (9)	0.0086 (8)	0.0064 (7)	0.0046 (8)
C51	0.0133 (8)	0.0264 (9)	0.0173 (8)	0.0048 (7)	0.0046 (7)	0.0066 (7)
C52	0.0291 (11)	0.0359 (12)	0.0319 (11)	0.0050 (9)	0.0084 (9)	−0.0106 (9)
C61	0.0646 (18)	0.0433 (14)	0.0188 (11)	0.0173 (13)	−0.0060 (11)	−0.0061 (10)
C62	0.0201 (9)	0.0229 (9)	0.0195 (9)	0.0061 (7)	0.0057 (7)	0.0006 (7)
C63	0.0237 (10)	0.0277 (10)	0.0207 (9)	0.0078 (8)	0.0058 (8)	0.0001 (8)
C64	0.0257 (10)	0.0277 (10)	0.0261 (10)	0.0035 (8)	0.0080 (8)	−0.0053 (8)
C65	0.0299 (11)	0.0222 (10)	0.0343 (11)	0.0043 (8)	0.0125 (9)	0.0009 (8)
C66	0.0260 (10)	0.0257 (10)	0.0293 (10)	0.0068 (8)	0.0101 (8)	0.0068 (8)
C67	0.0194 (9)	0.0236 (9)	0.0230 (9)	0.0059 (7)	0.0076 (7)	0.0031 (7)
C68	0.0214 (9)	0.0270 (10)	0.0209 (9)	0.0097 (8)	0.0063 (7)	0.0071 (7)
C69	0.0184 (9)	0.0316 (10)	0.0173 (9)	0.0069 (8)	0.0030 (7)	0.0033 (8)
C70	0.0281 (11)	0.0350 (11)	0.0241 (10)	0.0106 (9)	0.0016 (8)	0.0074 (9)
C71	0.0327 (12)	0.0464 (14)	0.0218 (10)	0.0137 (10)	−0.0020 (9)	0.0083 (9)
C72	0.0258 (11)	0.0457 (13)	0.0197 (10)	0.0089 (9)	−0.0015 (8)	−0.0002 (9)
C73	0.0214 (10)	0.0351 (11)	0.0208 (9)	0.0075 (8)	0.0019 (8)	−0.0011 (8)
C74	0.0174 (9)	0.0315 (10)	0.0182 (9)	0.0071 (8)	0.0036 (7)	0.0027 (8)
C75	0.0231 (10)	0.0290 (10)	0.0194 (9)	0.0057 (8)	0.0036 (7)	−0.0010 (8)
C76	0.0245 (10)	0.0251 (10)	0.0240 (10)	0.0075 (8)	0.0059 (8)	0.0019 (8)
C77	0.0328 (11)	0.0277 (11)	0.0289 (11)	0.0086 (9)	0.0056 (9)	−0.0025 (8)
C78	0.0386 (13)	0.0240 (10)	0.0405 (13)	0.0118 (9)	0.0082 (10)	0.0005 (9)
C79	0.0308 (11)	0.0266 (10)	0.0344 (11)	0.0105 (9)	0.0060 (9)	0.0090 (9)
C80	0.0251 (10)	0.0254 (10)	0.0252 (10)	0.0056 (8)	0.0034 (8)	0.0052 (8)
C81	0.0238 (10)	0.0235 (10)	0.0243 (10)	0.0068 (8)	0.0048 (8)	0.0040 (8)
C82	0.0391 (13)	0.0318 (12)	0.0307 (12)	0.0076 (10)	−0.0074 (10)	0.0110 (9)
C91	0.0268 (10)	0.0187 (9)	0.0299 (10)	0.0098 (8)	0.0077 (8)	0.0036 (8)
C92	0.0475 (15)	0.0749 (19)	0.0237 (11)	0.0415 (14)	0.0138 (10)	0.0202 (12)
C93	0.0250 (12)	0.0307 (13)	0.108 (3)	0.0047 (10)	0.0126 (14)	−0.0171 (15)
C101	0.0227 (10)	0.0243 (10)	0.0259 (10)	0.0037 (8)	0.0061 (8)	0.0016 (8)
C102	0.068 (2)	0.0219 (12)	0.081 (2)	0.0044 (12)	0.0373 (17)	0.0059 (13)
C103	0.0339 (14)	0.0588 (18)	0.0582 (18)	−0.0103 (12)	0.0206 (13)	0.0121 (14)
C110	0.0610 (18)	0.0378 (14)	0.0460 (16)	0.0085 (13)	0.0150 (13)	0.0151 (12)
C111	0.055 (2)	0.120 (3)	0.063 (2)	0.011 (2)	0.0313 (18)	0.032 (2)
C112	0.063 (2)	0.0597 (19)	0.0393 (15)	−0.0102 (15)	0.0151 (14)	0.0039 (13)
C121	0.0257 (10)	0.0320 (11)	0.0178 (9)	0.0088 (8)	0.0072 (8)	0.0050 (8)
C131	0.0194 (9)	0.0208 (9)	0.0205 (9)	0.0063 (7)	0.0046 (7)	0.0026 (7)
C141	0.0299 (11)	0.0188 (9)	0.0167 (9)	0.0037 (8)	0.0019 (8)	0.0025 (7)
C151	0.0466 (14)	0.0459 (14)	0.0160 (9)	0.0193 (11)	0.0061 (9)	0.0082 (9)
C161	0.058 (5)	0.035 (4)	0.0200 (16)	0.028 (3)	0.004 (3)	0.006 (2)
C961	0.023 (5)	0.012 (4)	0.0205 (18)	0.001 (3)	0.000 (3)	0.004 (2)
N1	0.0218 (8)	0.0227 (8)	0.0208 (8)	0.0079 (6)	0.0090 (6)	0.0104 (6)
N2	0.0185 (8)	0.0181 (7)	0.0229 (8)	0.0034 (6)	0.0060 (6)	0.0082 (6)
N31	0.0142 (7)	0.0173 (7)	0.0264 (8)	0.0021 (6)	0.0060 (6)	0.0021 (6)
N32	0.0154 (7)	0.0196 (8)	0.0230 (8)	0.0003 (6)	0.0061 (6)	−0.0034 (6)
N51	0.0190 (8)	0.0264 (8)	0.0175 (7)	0.0057 (6)	0.0025 (6)	0.0019 (6)
N52	0.0186 (8)	0.0264 (8)	0.0173 (7)	0.0066 (6)	0.0032 (6)	0.0039 (6)
N91	0.0232 (9)	0.0289 (9)	0.0323 (10)	0.0107 (7)	0.0026 (7)	−0.0065 (7)
N101	0.0271 (9)	0.0283 (9)	0.0315 (9)	−0.0031 (7)	0.0113 (8)	0.0045 (7)
N110	0.0518 (14)	0.0428 (12)	0.0386 (12)	0.0103 (10)	0.0170 (10)	0.0082 (9)
N121	0.0223 (9)	0.0261 (9)	0.0289 (9)	0.0065 (7)	0.0105 (7)	0.0110 (7)
N131	0.0180 (8)	0.0192 (8)	0.0199 (8)	0.0015 (6)	0.0050 (6)	0.0013 (6)
N141	0.0318 (10)	0.0248 (9)	0.0227 (8)	0.0073 (7)	0.0092 (7)	0.0053 (7)
N151	0.0349 (10)	0.0315 (10)	0.0240 (9)	0.0115 (8)	0.0014 (8)	0.0066 (7)
N161	0.0470 (12)	0.0336 (10)	0.0224 (8)	0.0123 (9)	0.0039 (8)	0.0024 (7)
O1	0.0206 (6)	0.0190 (6)	0.0178 (6)	0.0053 (5)	0.0029 (5)	0.0052 (5)
O2	0.0179 (6)	0.0157 (6)	0.0153 (6)	0.0012 (5)	0.0044 (5)	0.0028 (5)
O3	0.0223 (6)	0.0178 (6)	0.0129 (6)	0.0039 (5)	0.0056 (5)	0.0038 (5)
O4	0.0261 (7)	0.0173 (6)	0.0156 (6)	0.0064 (5)	0.0066 (5)	0.0033 (5)
O31	0.0312 (7)	0.0205 (7)	0.0172 (6)	0.0104 (6)	0.0088 (5)	0.0076 (5)
O32	0.0177 (6)	0.0195 (6)	0.0130 (6)	0.0040 (5)	0.0043 (5)	0.0035 (5)
O33	0.0178 (6)	0.0226 (6)	0.0152 (6)	0.0058 (5)	0.0042 (5)	0.0040 (5)
O34	0.0266 (7)	0.0283 (7)	0.0208 (7)	0.0098 (6)	0.0054 (6)	−0.0005 (6)
O61	0.0418 (9)	0.0290 (8)	0.0188 (7)	0.0112 (7)	−0.0050 (6)	−0.0029 (6)
O62	0.0295 (8)	0.0216 (7)	0.0193 (7)	0.0064 (6)	−0.0015 (6)	0.0023 (5)
O63	0.0347 (8)	0.0217 (7)	0.0203 (7)	0.0092 (6)	−0.0016 (6)	0.0023 (5)
O64	0.0382 (9)	0.0243 (7)	0.0240 (7)	0.0072 (6)	−0.0024 (6)	0.0073 (6)
O91	0.0223 (7)	0.0218 (7)	0.0211 (7)	0.0077 (5)	0.0059 (5)	0.0050 (5)
O101	0.0267 (7)	0.0197 (7)	0.0252 (7)	0.0010 (5)	0.0093 (6)	0.0047 (5)
O110	0.0822 (17)	0.0565 (14)	0.0609 (15)	0.0144 (12)	0.0128 (13)	0.0216 (11)
O971	0.032 (3)	0.026 (3)	0.031 (3)	0.004 (2)	0.004 (2)	0.001 (2)
S121	0.0194 (3)	0.0920 (6)	0.0300 (3)	0.0114 (3)	0.0091 (2)	−0.0001 (3)
S131	0.0325 (3)	0.0404 (3)	0.0586 (4)	−0.0051 (3)	0.0200 (3)	0.0198 (3)
S141	0.0280 (3)	0.0428 (3)	0.0449 (3)	0.0059 (2)	0.0148 (3)	0.0029 (3)
S151	0.0513 (5)	0.1423 (9)	0.0340 (4)	0.0500 (5)	0.0135 (3)	0.0104 (5)
S161	0.1116 (17)	0.0738 (13)	0.0242 (5)	0.0725 (14)	0.0286 (7)	0.0186 (6)
S961	0.0349 (12)	0.0298 (12)	0.0178 (8)	0.0150 (10)	0.0082 (7)	0.0042 (7)
Sm1	0.01849 (5)	0.01405 (4)	0.01404 (4)	0.00403 (3)	0.00687 (3)	0.00452 (3)
Zn1	0.01724 (10)	0.01703 (10)	0.01765 (10)	0.00387 (8)	0.00633 (8)	0.00702 (8)
Zn31	0.01394 (10)	0.01608 (10)	0.01577 (10)	0.00152 (7)	0.00384 (7)	0.00109 (7)
Zn61	0.03172 (13)	0.02864 (12)	0.01707 (11)	0.00918 (10)	−0.00126 (9)	0.00330 (9)
Zn62	0.02403 (11)	0.02136 (11)	0.01524 (10)	0.00642 (9)	0.00265 (8)	0.00237 (8)

### Geometric parameters (Å, º)

**Table:**

C1—H1A	0.9800	C67—C68	1.452 (3)
C1—H1B	0.9800	C68—H68	0.9500
C1—H1C	0.9800	C68—N52	1.284 (3)
C1—O1	1.431 (2)	C69—C70	1.396 (3)
C2—C3	1.421 (2)	C69—C74	1.408 (3)
C2—C7	1.415 (3)	C69—N52	1.417 (2)
C2—O2	1.329 (2)	C70—H70	0.9500
C3—C4	1.379 (3)	C70—C71	1.386 (3)
C3—O1	1.367 (2)	C71—H71	0.9500
C4—H4	0.9500	C71—C72	1.386 (3)
C4—C5	1.400 (3)	C72—H72	0.9500
C5—H5	0.9500	C72—C73	1.379 (3)
C5—C6	1.367 (3)	C73—H73	0.9500
C6—H6	0.9500	C73—C74	1.396 (3)
C6—C7	1.415 (3)	C74—N51	1.418 (2)
C7—C8	1.444 (3)	C75—H75	0.9500
C8—H8	0.9500	C75—C76	1.451 (3)
C8—N2	1.285 (3)	C75—N51	1.287 (3)
C9—C10	1.400 (3)	C76—C77	1.417 (3)
C9—C14	1.405 (3)	C76—C81	1.410 (3)
C9—N2	1.420 (2)	C77—H77	0.9500
C10—H10	0.9500	C77—C78	1.369 (3)
C10—C11	1.380 (3)	C78—H78	0.9500
C11—H11	0.9500	C78—C79	1.400 (3)
C11—C12	1.384 (4)	C79—H79	0.9500
C12—H12	0.9500	C79—C80	1.374 (3)
C12—C13	1.386 (3)	C80—C81	1.420 (3)
C13—H13	0.9500	C80—O64	1.365 (3)
C13—C14	1.397 (3)	C81—O63	1.317 (2)
C14—N1	1.417 (2)	C82—H82A	0.9800
C15—H15	0.9500	C82—H82B	0.9800
C15—C16	1.445 (3)	C82—H82C	0.9800
C15—N1	1.283 (3)	C82—O64	1.421 (2)
C16—C17	1.412 (3)	C91—H91	0.9500
C16—C21	1.411 (3)	C91—N91	1.317 (3)
C17—H17	0.9500	C91—O91	1.241 (2)
C17—C18	1.369 (3)	C92—H92A	0.9800
C18—H18	0.9500	C92—H92B	0.9800
C18—C19	1.402 (3)	C92—H92C	0.9800
C19—H19	0.9500	C92—N91	1.452 (3)
C19—C20	1.378 (3)	C93—H93A	0.9800
C20—C21	1.406 (3)	C93—H93B	0.9800
C20—O4	1.381 (2)	C93—H93C	0.9800
C21—O3	1.333 (2)	C93—N91	1.451 (3)
C22—H22A	0.9800	C101—H101	0.9500
C22—H22B	0.9800	C101—N101	1.305 (3)
C22—H22C	0.9800	C101—O101	1.243 (2)
C22—O4	1.436 (2)	C102—H10A	0.9800
C31—H31A	0.9800	C102—H10B	0.9800
C31—H31B	0.9800	C102—H10C	0.9800
C31—H31C	0.9800	C102—N101	1.446 (3)
C31—O31	1.429 (2)	C103—H10D	0.9800
C32—C33	1.409 (3)	C103—H10E	0.9800
C32—C37	1.415 (3)	C103—H10F	0.9800
C32—O32	1.321 (2)	C103—N101	1.461 (3)
C33—C34	1.378 (3)	C110—H110	0.9500
C33—O31	1.376 (2)	C110—N110	1.347 (4)
C34—H34	0.9500	C110—O110	1.235 (4)
C34—C35	1.402 (3)	C111—H11A	0.9800
C35—H35	0.9500	C111—H11B	0.9800
C35—C36	1.367 (3)	C111—H11C	0.9800
C36—H36	0.9500	C111—N110	1.424 (4)
C36—C37	1.416 (3)	C112—H11D	0.9800
C37—C38	1.449 (3)	C112—H11E	0.9800
C38—H38	0.9500	C112—H11F	0.9800
C38—N32	1.282 (3)	C112—N110	1.437 (4)
C39—C40	1.401 (3)	C121—N121	1.159 (3)
C39—C44	1.401 (3)	C121—S121	1.626 (2)
C39—N32	1.417 (2)	C131—N131	1.157 (3)
C40—H40	0.9500	C131—S131	1.620 (2)
C40—C41	1.383 (3)	C141—N141	1.158 (3)
C41—H41	0.9500	C141—S141	1.619 (2)
C41—C42	1.377 (4)	C151—N151	1.150 (3)
C42—H42	0.9500	C151—S151	1.624 (3)
C42—C43	1.385 (3)	C161—N161	1.203 (7)
C43—H43	0.9500	C161—S161	1.635 (6)
C43—C44	1.399 (3)	C961—N161	1.070 (14)
C44—N31	1.424 (2)	C961—S961	1.626 (13)
C45—H45	0.9500	N1—Zn1	2.0863 (16)
C45—C46	1.442 (3)	N2—Zn1	2.0451 (16)
C45—N31	1.286 (3)	N31—Zn31	2.0534 (16)
C46—C47	1.416 (3)	N32—Zn31	2.0644 (16)
C46—C51	1.414 (3)	N51—Zn62	2.0508 (16)
C47—H47	0.9500	N52—Zn62	2.0486 (16)
C47—C48	1.366 (3)	N121—Zn1	1.9842 (17)
C48—H48	0.9500	N131—Zn31	1.9786 (16)
C48—C49	1.399 (3)	N141—Zn62	1.9687 (18)
C49—H49	0.9500	N151—Zn61	1.949 (2)
C49—C50	1.382 (3)	N161—Zn61	1.943 (2)
C50—C51	1.412 (3)	O2—Sm1	2.4417 (12)
C50—O34	1.366 (2)	O2—Zn1	2.0395 (12)
C51—O33	1.329 (2)	O3—Sm1	2.3348 (12)
C52—H52A	0.9800	O3—Zn1	1.9985 (13)
C52—H52B	0.9800	O4—Sm1	2.6707 (13)
C52—H52C	0.9800	O31—Sm1	2.6934 (13)
C52—O34	1.432 (2)	O32—Sm1	2.3599 (12)
C61—H61A	0.9800	O32—Zn31	2.0143 (13)
C61—H61B	0.9800	O33—Sm1	2.4038 (13)
C61—H61C	0.9800	O33—Zn31	2.0175 (13)
C61—O61	1.418 (3)	O62—Zn61	2.0267 (14)
C62—C63	1.419 (3)	O62—Zn62	2.0132 (14)
C62—C67	1.408 (3)	O63—Zn61	2.0135 (14)
C62—O62	1.321 (2)	O63—Zn62	2.0073 (14)
C63—C64	1.372 (3)	O91—Sm1	2.3831 (13)
C63—O61	1.369 (3)	O101—Sm1	2.3476 (13)
C64—H64	0.9500	O971—H97A	0.8700
C64—C65	1.399 (3)	O971—H97B	0.8699
C65—H65	0.9500	Sm1—Zn1	3.5372 (2)
C65—C66	1.373 (3)	Sm1—Zn31	3.5444 (2)
C66—H66	0.9500	Zn61—Zn62	3.1201 (3)
C66—C67	1.414 (3)		
			
H1A—C1—H1B	109.5	N91—C91—H91	117.8
H1A—C1—H1C	109.5	O91—C91—H91	117.8
H1B—C1—H1C	109.5	O91—C91—N91	124.34 (19)
O1—C1—H1A	109.5	H92A—C92—H92B	109.5
O1—C1—H1B	109.5	H92A—C92—H92C	109.5
O1—C1—H1C	109.5	H92B—C92—H92C	109.5
C7—C2—C3	117.35 (16)	N91—C92—H92A	109.5
O2—C2—C3	118.66 (16)	N91—C92—H92B	109.5
O2—C2—C7	123.99 (16)	N91—C92—H92C	109.5
C4—C3—C2	121.73 (17)	H93A—C93—H93B	109.5
O1—C3—C2	113.53 (16)	H93A—C93—H93C	109.5
O1—C3—C4	124.74 (17)	H93B—C93—H93C	109.5
C3—C4—H4	120.0	N91—C93—H93A	109.5
C3—C4—C5	120.00 (18)	N91—C93—H93B	109.5
C5—C4—H4	120.0	N91—C93—H93C	109.5
C4—C5—H5	120.1	N101—C101—H101	117.9
C6—C5—C4	119.80 (18)	O101—C101—H101	117.9
C6—C5—H5	120.1	O101—C101—N101	124.2 (2)
C5—C6—H6	119.3	H10A—C102—H10B	109.5
C5—C6—C7	121.31 (18)	H10A—C102—H10C	109.5
C7—C6—H6	119.3	H10B—C102—H10C	109.5
C2—C7—C8	124.65 (17)	N101—C102—H10A	109.5
C6—C7—C2	119.60 (17)	N101—C102—H10B	109.5
C6—C7—C8	115.71 (17)	N101—C102—H10C	109.5
C7—C8—H8	117.3	H10D—C103—H10E	109.5
N2—C8—C7	125.39 (17)	H10D—C103—H10F	109.5
N2—C8—H8	117.3	H10E—C103—H10F	109.5
C10—C9—C14	120.05 (18)	N101—C103—H10D	109.5
C10—C9—N2	123.68 (19)	N101—C103—H10E	109.5
C14—C9—N2	116.17 (17)	N101—C103—H10F	109.5
C9—C10—H10	120.1	N110—C110—H110	117.8
C11—C10—C9	119.8 (2)	O110—C110—H110	117.8
C11—C10—H10	120.1	O110—C110—N110	124.4 (3)
C10—C11—H11	119.8	H11A—C111—H11B	109.5
C10—C11—C12	120.3 (2)	H11A—C111—H11C	109.5
C12—C11—H11	119.8	H11B—C111—H11C	109.5
C11—C12—H12	119.7	N110—C111—H11A	109.5
C11—C12—C13	120.6 (2)	N110—C111—H11B	109.5
C13—C12—H12	119.7	N110—C111—H11C	109.5
C12—C13—H13	120.0	H11D—C112—H11E	109.5
C12—C13—C14	120.1 (2)	H11D—C112—H11F	109.5
C14—C13—H13	120.0	H11E—C112—H11F	109.5
C9—C14—N1	115.65 (17)	N110—C112—H11D	109.5
C13—C14—C9	119.10 (19)	N110—C112—H11E	109.5
C13—C14—N1	125.23 (19)	N110—C112—H11F	109.5
C16—C15—H15	117.6	N121—C121—S121	178.9 (2)
N1—C15—H15	117.6	N131—C131—S131	178.92 (19)
N1—C15—C16	124.87 (17)	N141—C141—S141	179.4 (2)
C17—C16—C15	117.83 (17)	N151—C151—S151	177.7 (2)
C21—C16—C15	123.11 (17)	N161—C161—S161	179.4 (8)
C21—C16—C17	118.86 (18)	N161—C961—S961	177.1 (16)
C16—C17—H17	119.4	C14—N1—Zn1	111.82 (13)
C18—C17—C16	121.11 (18)	C15—N1—C14	123.18 (17)
C18—C17—H17	119.4	C15—N1—Zn1	124.87 (13)
C17—C18—H18	119.7	C8—N2—C9	121.74 (17)
C17—C18—C19	120.54 (18)	C8—N2—Zn1	125.65 (13)
C19—C18—H18	119.7	C9—N2—Zn1	112.50 (13)
C18—C19—H19	120.5	C44—N31—Zn31	112.56 (13)
C20—C19—C18	118.94 (18)	C45—N31—C44	122.48 (17)
C20—C19—H19	120.5	C45—N31—Zn31	124.93 (14)
C19—C20—C21	121.94 (17)	C38—N32—C39	123.60 (17)
C19—C20—O4	125.38 (17)	C38—N32—Zn31	123.97 (14)
O4—C20—C21	112.63 (15)	C39—N32—Zn31	112.41 (13)
C20—C21—C16	118.57 (17)	C74—N51—Zn62	110.35 (13)
O3—C21—C16	124.27 (17)	C75—N51—C74	122.96 (17)
O3—C21—C20	117.06 (16)	C75—N51—Zn62	126.57 (14)
H22A—C22—H22B	109.5	C68—N52—C69	123.05 (17)
H22A—C22—H22C	109.5	C68—N52—Zn62	126.59 (14)
H22B—C22—H22C	109.5	C69—N52—Zn62	110.33 (13)
O4—C22—H22A	109.5	C91—N91—C92	121.3 (2)
O4—C22—H22B	109.5	C91—N91—C93	122.6 (2)
O4—C22—H22C	109.5	C93—N91—C92	116.1 (2)
H31A—C31—H31B	109.5	C101—N101—C102	121.1 (2)
H31A—C31—H31C	109.5	C101—N101—C103	122.1 (2)
H31B—C31—H31C	109.5	C102—N101—C103	116.8 (2)
O31—C31—H31A	109.5	C110—N110—C111	121.5 (3)
O31—C31—H31B	109.5	C110—N110—C112	119.5 (2)
O31—C31—H31C	109.5	C111—N110—C112	118.9 (3)
C33—C32—C37	118.09 (17)	C121—N121—Zn1	176.12 (18)
O32—C32—C33	117.14 (16)	C131—N131—Zn31	172.86 (16)
O32—C32—C37	124.68 (17)	C141—N141—Zn62	175.46 (17)
C34—C33—C32	122.15 (18)	C151—N151—Zn61	173.9 (2)
O31—C33—C32	112.33 (15)	C161—N161—Zn61	170.9 (5)
O31—C33—C34	125.46 (18)	C961—N161—Zn61	164.2 (7)
C33—C34—H34	120.4	C3—O1—C1	116.75 (15)
C33—C34—C35	119.25 (19)	C2—O2—Sm1	130.20 (11)
C35—C34—H34	120.4	C2—O2—Zn1	122.79 (11)
C34—C35—H35	120.0	Zn1—O2—Sm1	103.89 (5)
C36—C35—C34	120.03 (18)	C21—O3—Sm1	127.23 (11)
C36—C35—H35	120.0	C21—O3—Zn1	123.57 (11)
C35—C36—H36	119.2	Zn1—O3—Sm1	109.18 (5)
C35—C36—C37	121.59 (19)	C20—O4—C22	116.69 (14)
C37—C36—H36	119.2	C20—O4—Sm1	115.77 (10)
C32—C37—C36	118.72 (18)	C22—O4—Sm1	126.89 (11)
C32—C37—C38	123.00 (17)	C31—O31—Sm1	124.14 (11)
C36—C37—C38	118.06 (18)	C33—O31—C31	117.42 (15)
C37—C38—H38	118.0	C33—O31—Sm1	118.41 (11)
N32—C38—C37	124.09 (17)	C32—O32—Sm1	130.47 (11)
N32—C38—H38	118.0	C32—O32—Zn31	120.58 (11)
C40—C39—C44	119.82 (19)	Zn31—O32—Sm1	107.99 (5)
C40—C39—N32	124.1 (2)	C51—O33—Sm1	130.64 (11)
C44—C39—N32	116.04 (17)	C51—O33—Zn31	122.08 (11)
C39—C40—H40	120.2	Zn31—O33—Sm1	106.25 (5)
C41—C40—C39	119.6 (2)	C50—O34—C52	116.72 (17)
C41—C40—H40	120.2	C63—O61—C61	117.58 (18)
C40—C41—H41	119.7	C62—O62—Zn61	132.34 (12)
C42—C41—C40	120.6 (2)	C62—O62—Zn62	125.95 (12)
C42—C41—H41	119.7	Zn62—O62—Zn61	101.13 (6)
C41—C42—H42	119.6	C81—O63—Zn61	129.26 (13)
C41—C42—C43	120.7 (2)	C81—O63—Zn62	128.36 (13)
C43—C42—H42	119.6	Zn62—O63—Zn61	101.79 (6)
C42—C43—H43	120.2	C80—O64—C82	118.24 (17)
C42—C43—C44	119.6 (2)	C91—O91—Sm1	131.76 (12)
C44—C43—H43	120.2	C101—O101—Sm1	136.34 (13)
C39—C44—N31	116.47 (17)	H97A—O971—H97B	109.5
C43—C44—C39	119.62 (19)	O2—Sm1—O4	119.60 (4)
C43—C44—N31	123.9 (2)	O2—Sm1—O31	111.28 (4)
C46—C45—H45	117.3	O2—Sm1—Zn1	34.04 (3)
N31—C45—H45	117.3	O2—Sm1—Zn31	75.83 (3)
N31—C45—C46	125.38 (18)	O3—Sm1—O2	66.10 (4)
C47—C46—C45	116.73 (18)	O3—Sm1—O4	60.69 (4)
C51—C46—C45	124.06 (17)	O3—Sm1—O31	159.39 (4)
C51—C46—C47	119.19 (19)	O3—Sm1—O32	134.80 (4)
C46—C47—H47	119.5	O3—Sm1—O33	80.48 (4)
C48—C47—C46	120.9 (2)	O3—Sm1—O91	89.74 (5)
C48—C47—H47	119.5	O3—Sm1—O101	119.90 (5)
C47—C48—H48	119.9	O3—Sm1—Zn1	32.25 (3)
C47—C48—C49	120.27 (19)	O3—Sm1—Zn31	108.18 (3)
C49—C48—H48	119.9	O4—Sm1—O31	108.63 (4)
C48—C49—H49	120.0	O4—Sm1—Zn1	90.48 (3)
C50—C49—C48	119.9 (2)	O4—Sm1—Zn31	146.28 (3)
C50—C49—H49	120.0	O31—Sm1—Zn1	142.97 (3)
C49—C50—C51	121.15 (19)	O31—Sm1—Zn31	90.16 (3)
O34—C50—C49	125.60 (19)	O32—Sm1—O2	78.61 (4)
O34—C50—C51	113.25 (16)	O32—Sm1—O4	161.77 (4)
C50—C51—C46	118.34 (17)	O32—Sm1—O31	59.85 (4)
O33—C51—C46	123.33 (17)	O32—Sm1—O33	65.80 (4)
O33—C51—C50	118.33 (17)	O32—Sm1—O91	114.01 (4)
H52A—C52—H52B	109.5	O32—Sm1—Zn1	107.06 (3)
H52A—C52—H52C	109.5	O32—Sm1—Zn31	32.72 (3)
H52B—C52—H52C	109.5	O33—Sm1—O2	79.92 (4)
O34—C52—H52A	109.5	O33—Sm1—O4	115.61 (4)
O34—C52—H52B	109.5	O33—Sm1—O31	119.80 (4)
O34—C52—H52C	109.5	O33—Sm1—Zn1	75.67 (3)
H61A—C61—H61B	109.5	O33—Sm1—Zn31	33.13 (3)
H61A—C61—H61C	109.5	O91—Sm1—O2	83.29 (4)
H61B—C61—H61C	109.5	O91—Sm1—O4	70.36 (4)
O61—C61—H61A	109.5	O91—Sm1—O31	69.71 (4)
O61—C61—H61B	109.5	O91—Sm1—O33	162.88 (5)
O61—C61—H61C	109.5	O91—Sm1—Zn1	88.55 (3)
C67—C62—C63	118.03 (18)	O91—Sm1—Zn31	143.36 (3)
O62—C62—C63	117.98 (17)	O101—Sm1—O2	167.46 (5)
O62—C62—C67	123.98 (17)	O101—Sm1—O4	71.53 (4)
C64—C63—C62	121.73 (19)	O101—Sm1—O31	67.27 (5)
O61—C63—C62	112.25 (18)	O101—Sm1—O32	90.46 (5)
O61—C63—C64	126.02 (19)	O101—Sm1—O33	90.05 (5)
C63—C64—H64	120.2	O101—Sm1—O91	107.03 (5)
C63—C64—C65	119.57 (19)	O101—Sm1—Zn1	149.69 (4)
C65—C64—H64	120.2	O101—Sm1—Zn31	91.66 (4)
C64—C65—H65	119.9	Zn1—Sm1—Zn31	90.491 (5)
C66—C65—C64	120.1 (2)	N1—Zn1—Sm1	117.86 (5)
C66—C65—H65	119.9	N2—Zn1—N1	79.60 (6)
C65—C66—H66	119.4	N2—Zn1—Sm1	118.28 (4)
C65—C66—C67	121.1 (2)	N121—Zn1—N1	108.05 (7)
C67—C66—H66	119.4	N121—Zn1—N2	112.95 (7)
C62—C67—C66	119.13 (18)	N121—Zn1—O2	104.31 (6)
C62—C67—C68	123.64 (18)	N121—Zn1—O3	108.25 (6)
C66—C67—C68	117.22 (18)	N121—Zn1—Sm1	115.01 (5)
C67—C68—H68	117.6	O2—Zn1—N1	147.54 (6)
N52—C68—C67	124.86 (18)	O2—Zn1—N2	90.27 (6)
N52—C68—H68	117.6	O2—Zn1—Sm1	42.08 (4)
C70—C69—C74	119.76 (19)	O3—Zn1—N1	87.12 (6)
C70—C69—N52	124.25 (19)	O3—Zn1—N2	138.79 (6)
C74—C69—N52	115.97 (17)	O3—Zn1—O2	80.40 (5)
C69—C70—H70	120.2	O3—Zn1—Sm1	38.57 (4)
C71—C70—C69	119.7 (2)	N31—Zn31—N32	80.42 (7)
C71—C70—H70	120.2	N31—Zn31—Sm1	116.69 (4)
C70—C71—H71	119.8	N32—Zn31—Sm1	120.54 (4)
C72—C71—C70	120.5 (2)	N131—Zn31—N31	120.99 (6)
C72—C71—H71	119.8	N131—Zn31—N32	104.40 (6)
C71—C72—H72	119.7	N131—Zn31—O32	102.67 (6)
C73—C72—C71	120.5 (2)	N131—Zn31—O33	105.95 (6)
C73—C72—H72	119.7	N131—Zn31—Sm1	110.51 (5)
C72—C73—H73	120.0	O32—Zn31—N31	136.30 (6)
C72—C73—C74	120.0 (2)	O32—Zn31—N32	87.56 (6)
C74—C73—H73	120.0	O32—Zn31—O33	79.86 (5)
C69—C74—N51	116.15 (17)	O32—Zn31—Sm1	39.29 (3)
C73—C74—C69	119.59 (18)	O33—Zn31—N31	89.29 (6)
C73—C74—N51	124.22 (19)	O33—Zn31—N32	149.00 (6)
C76—C75—H75	117.3	O33—Zn31—Sm1	40.63 (4)
N51—C75—H75	117.3	N151—Zn61—O62	114.80 (7)
N51—C75—C76	125.49 (18)	N151—Zn61—O63	112.60 (7)
C77—C76—C75	117.14 (19)	N151—Zn61—Zn62	129.02 (5)
C81—C76—C75	124.22 (18)	N161—Zn61—N151	119.03 (8)
C81—C76—C77	118.60 (19)	N161—Zn61—O62	112.66 (7)
C76—C77—H77	119.3	N161—Zn61—O63	113.62 (8)
C78—C77—C76	121.4 (2)	N161—Zn61—Zn62	111.94 (6)
C78—C77—H77	119.3	O62—Zn61—Zn62	39.28 (4)
C77—C78—H78	119.9	O63—Zn61—O62	76.86 (6)
C77—C78—C79	120.2 (2)	O63—Zn61—Zn62	39.03 (4)
C79—C78—H78	119.9	N51—Zn62—Zn61	123.45 (5)
C78—C79—H79	120.2	N52—Zn62—N51	80.67 (7)
C80—C79—C78	119.5 (2)	N52—Zn62—Zn61	125.32 (5)
C80—C79—H79	120.2	N141—Zn62—N51	110.27 (7)
C79—C80—C81	121.6 (2)	N141—Zn62—N52	106.67 (7)
O64—C80—C79	126.19 (19)	N141—Zn62—O62	111.31 (7)
O64—C80—C81	112.22 (18)	N141—Zn62—O63	108.59 (7)
C76—C81—C80	118.54 (19)	N141—Zn62—Zn61	107.65 (5)
O63—C81—C76	123.47 (18)	O62—Zn62—N51	138.41 (6)
O63—C81—C80	117.98 (18)	O62—Zn62—N52	88.34 (6)
H82A—C82—H82B	109.5	O62—Zn62—Zn61	39.59 (4)
H82A—C82—H82C	109.5	O63—Zn62—N51	88.94 (6)
H82B—C82—H82C	109.5	O63—Zn62—N52	144.69 (7)
O64—C82—H82A	109.5	O63—Zn62—O62	77.30 (6)
O64—C82—H82B	109.5	O63—Zn62—Zn61	39.18 (4)
O64—C82—H82C	109.5		
			
C2—C3—C4—C5	0.6 (3)	C49—C50—C51—C46	2.9 (3)
C2—C3—O1—C1	175.17 (15)	C49—C50—C51—O33	−176.88 (18)
C2—C7—C8—N2	−13.1 (3)	C49—C50—O34—C52	−13.0 (3)
C3—C2—C7—C6	−5.3 (2)	C50—C51—O33—Sm1	48.7 (2)
C3—C2—C7—C8	172.50 (16)	C50—C51—O33—Zn31	−144.55 (14)
C3—C2—O2—Sm1	53.4 (2)	C51—C46—C47—C48	3.9 (3)
C3—C2—O2—Zn1	−149.98 (13)	C51—C50—O34—C52	167.24 (17)
C3—C4—C5—C6	−2.0 (3)	C62—C63—C64—C65	−3.9 (3)
C4—C3—O1—C1	−4.9 (3)	C62—C63—O61—C61	−166.0 (2)
C4—C5—C6—C7	−0.3 (3)	C62—C67—C68—N52	15.0 (3)
C5—C6—C7—C2	4.1 (3)	C63—C62—C67—C66	−2.9 (3)
C5—C6—C7—C8	−173.92 (17)	C63—C62—C67—C68	175.68 (18)
C6—C7—C8—N2	164.79 (18)	C63—C62—O62—Zn61	−10.8 (3)
C7—C2—C3—C4	3.1 (3)	C63—C62—O62—Zn62	158.78 (14)
C7—C2—C3—O1	−176.96 (15)	C63—C64—C65—C66	−1.3 (3)
C7—C2—O2—Sm1	−126.30 (15)	C64—C63—O61—C61	14.7 (3)
C7—C2—O2—Zn1	30.4 (2)	C64—C65—C66—C67	4.3 (3)
C7—C8—N2—C9	−176.64 (17)	C65—C66—C67—C62	−2.1 (3)
C7—C8—N2—Zn1	7.3 (3)	C65—C66—C67—C68	179.25 (19)
C9—C10—C11—C12	1.0 (4)	C66—C67—C68—N52	−166.43 (19)
C9—C14—N1—C15	170.83 (18)	C67—C62—C63—C64	5.9 (3)
C9—C14—N1—Zn1	−13.1 (2)	C67—C62—C63—O61	−173.43 (17)
C10—C9—C14—C13	−3.9 (3)	C67—C62—O62—Zn61	167.78 (14)
C10—C9—C14—N1	174.41 (19)	C67—C62—O62—Zn62	−22.6 (3)
C10—C9—N2—C8	23.7 (3)	C67—C68—N52—C69	−177.88 (18)
C10—C9—N2—Zn1	−159.72 (18)	C67—C68—N52—Zn62	−0.1 (3)
C10—C11—C12—C13	−2.4 (4)	C69—C70—C71—C72	0.1 (4)
C11—C12—C13—C14	0.6 (4)	C69—C74—N51—C75	−159.30 (19)
C12—C13—C14—C9	2.6 (3)	C69—C74—N51—Zn62	17.0 (2)
C12—C13—C14—N1	−175.6 (2)	C70—C69—C74—C73	1.4 (3)
C13—C14—N1—C15	−10.9 (3)	C70—C69—C74—N51	−176.47 (18)
C13—C14—N1—Zn1	165.10 (17)	C70—C69—N52—C68	−23.3 (3)
C14—C9—C10—C11	2.2 (4)	C70—C69—N52—Zn62	158.57 (17)
C14—C9—N2—C8	−160.00 (18)	C70—C71—C72—C73	0.6 (4)
C14—C9—N2—Zn1	16.6 (2)	C71—C72—C73—C74	−0.3 (3)
C15—C16—C17—C18	−175.54 (19)	C72—C73—C74—C69	−0.7 (3)
C15—C16—C21—C20	173.49 (18)	C72—C73—C74—N51	177.04 (19)
C15—C16—C21—O3	−2.8 (3)	C73—C74—N51—C75	22.9 (3)
C16—C15—N1—C14	−177.64 (18)	C73—C74—N51—Zn62	−160.84 (16)
C16—C15—N1—Zn1	6.9 (3)	C74—C69—C70—C71	−1.2 (3)
C16—C17—C18—C19	1.4 (3)	C74—C69—N52—C68	158.65 (19)
C16—C21—O3—Sm1	151.92 (14)	C74—C69—N52—Zn62	−19.5 (2)
C16—C21—O3—Zn1	−29.7 (2)	C75—C76—C77—C78	179.7 (2)
C17—C16—C21—C20	−1.3 (3)	C75—C76—C81—C80	−177.5 (2)
C17—C16—C21—O3	−177.59 (18)	C75—C76—C81—O63	2.0 (3)
C17—C18—C19—C20	−0.4 (3)	C76—C75—N51—C74	178.54 (19)
C18—C19—C20—C21	−1.5 (3)	C76—C75—N51—Zn62	2.9 (3)
C18—C19—C20—O4	175.67 (18)	C76—C77—C78—C79	−2.4 (4)
C19—C20—C21—C16	2.3 (3)	C76—C81—O63—Zn61	−153.77 (16)
C19—C20—C21—O3	178.87 (17)	C76—C81—O63—Zn62	15.7 (3)
C19—C20—O4—C22	10.7 (3)	C77—C76—C81—C80	0.0 (3)
C19—C20—O4—Sm1	−160.67 (15)	C77—C76—C81—O63	179.5 (2)
C20—C21—O3—Sm1	−24.4 (2)	C77—C78—C79—C80	0.7 (4)
C20—C21—O3—Zn1	153.97 (13)	C78—C79—C80—C81	1.4 (3)
C21—C16—C17—C18	−0.5 (3)	C78—C79—C80—O64	−179.9 (2)
C21—C20—O4—C22	−171.90 (16)	C79—C80—C81—C76	−1.7 (3)
C21—C20—O4—Sm1	16.70 (19)	C79—C80—C81—O63	178.8 (2)
C32—C33—C34—C35	−2.8 (3)	C79—C80—O64—C82	2.3 (3)
C32—C33—O31—C31	−176.21 (16)	C80—C81—O63—Zn61	25.7 (3)
C32—C33—O31—Sm1	5.8 (2)	C80—C81—O63—Zn62	−164.81 (15)
C32—C37—C38—N32	18.3 (3)	C81—C76—C77—C78	2.0 (3)
C33—C32—C37—C36	−3.0 (3)	C81—C80—O64—C82	−178.91 (19)
C33—C32—C37—C38	171.44 (17)	N1—C15—C16—C17	−170.43 (19)
C33—C32—O32—Sm1	−15.5 (2)	N1—C15—C16—C21	14.8 (3)
C33—C32—O32—Zn31	151.84 (13)	N2—C9—C10—C11	178.3 (2)
C33—C34—C35—C36	−1.0 (3)	N2—C9—C14—C13	179.61 (18)
C34—C33—O31—C31	6.4 (3)	N2—C9—C14—N1	−2.0 (3)
C34—C33—O31—Sm1	−171.61 (15)	N31—C45—C46—C47	163.73 (19)
C34—C35—C36—C37	2.6 (3)	N31—C45—C46—C51	−14.5 (3)
C35—C36—C37—C32	−0.6 (3)	N32—C39—C40—C41	177.57 (18)
C35—C36—C37—C38	−175.31 (19)	N32—C39—C44—C43	−176.90 (17)
C36—C37—C38—N32	−167.26 (19)	N32—C39—C44—N31	3.3 (2)
C37—C32—C33—C34	4.8 (3)	N51—C75—C76—C77	170.7 (2)
C37—C32—C33—O31	−172.72 (16)	N51—C75—C76—C81	−11.7 (3)
C37—C32—O32—Sm1	161.14 (13)	N52—C69—C70—C71	−179.1 (2)
C37—C32—O32—Zn31	−31.5 (2)	N52—C69—C74—C73	179.59 (18)
C37—C38—N32—C39	−173.79 (17)	N52—C69—C74—N51	1.7 (3)
C37—C38—N32—Zn31	7.9 (3)	N91—C91—O91—Sm1	159.59 (15)
C39—C40—C41—C42	−0.6 (3)	N101—C101—O101—Sm1	−165.90 (15)
C39—C44—N31—C45	−170.26 (18)	O1—C3—C4—C5	−179.35 (17)
C39—C44—N31—Zn31	7.9 (2)	O2—C2—C3—C4	−176.60 (16)
C40—C39—C44—C43	2.0 (3)	O2—C2—C3—O1	3.3 (2)
C40—C39—C44—N31	−177.82 (17)	O2—C2—C7—C6	174.33 (16)
C40—C39—N32—C38	−10.1 (3)	O2—C2—C7—C8	−7.8 (3)
C40—C39—N32—Zn31	168.42 (16)	O4—C20—C21—C16	−175.19 (16)
C40—C41—C42—C43	1.6 (3)	O4—C20—C21—O3	1.4 (2)
C41—C42—C43—C44	−0.8 (3)	O31—C33—C34—C35	174.34 (18)
C42—C43—C44—C39	−1.0 (3)	O32—C32—C33—C34	−178.39 (17)
C42—C43—C44—N31	178.85 (19)	O32—C32—C33—O31	4.1 (2)
C43—C44—N31—C45	9.9 (3)	O32—C32—C37—C36	−179.60 (17)
C43—C44—N31—Zn31	−171.95 (15)	O32—C32—C37—C38	−5.1 (3)
C44—C39—C40—C41	−1.3 (3)	O34—C50—C51—C46	−177.34 (16)
C44—C39—N32—C38	168.79 (18)	O34—C50—C51—O33	2.9 (2)
C44—C39—N32—Zn31	−12.7 (2)	O61—C63—C64—C65	175.4 (2)
C45—C46—C47—C48	−174.4 (2)	O62—C62—C63—C64	−175.42 (19)
C45—C46—C51—C50	173.08 (18)	O62—C62—C63—O61	5.2 (3)
C45—C46—C51—O33	−7.2 (3)	O62—C62—C67—C66	178.57 (18)
C46—C45—N31—C44	−178.56 (18)	O62—C62—C67—C68	−2.9 (3)
C46—C45—N31—Zn31	3.5 (3)	O64—C80—C81—C76	179.37 (18)
C46—C47—C48—C49	−0.4 (3)	O64—C80—C81—O63	−0.1 (3)
C46—C51—O33—Sm1	−131.01 (16)	O91—C91—N91—C92	−2.5 (3)
C46—C51—O33—Zn31	35.7 (2)	O91—C91—N91—C93	178.8 (2)
C47—C46—C51—C50	−5.1 (3)	O101—C101—N101—C102	0.1 (4)
C47—C46—C51—O33	174.67 (18)	O101—C101—N101—C103	−179.0 (2)
C47—C48—C49—C50	−1.9 (3)	O110—C110—N110—C111	−1.3 (5)
C48—C49—C50—C51	0.6 (3)	O110—C110—N110—C112	176.7 (3)
C48—C49—C50—O34	−179.11 (19)		
